# Direct Zinc Determination in Brazilian Sugar Cane Spirit by Solid-Phase Extraction Using *Moringa oleifera* Husks in a Flow System with Detection by FAAS

**DOI:** 10.1155/2011/765746

**Published:** 2011-07-07

**Authors:** Vanessa N. Alves, Simone S. O. Borges, Nivia M. M. Coelho

**Affiliations:** Instituto de Química, Universidade Federal de Uberlândia, Uberlândia 38400-902, MG, Brazil

## Abstract

This paper reports a method for the determination of zinc in Brazilian sugar cane spirit, (*cachaça* in Portuguese), using solid-phase extraction with a flow injection analysis system and detection by FAAS. The sorbent material used was activated carbon obtained from *Moringa oleifera* husks. Flow and chemical variables of the proposed system were optimized through multivariate designs. The factors selected were sorbent mass, sample pH, sample flow rate, and eluent concentration. The optimum extraction conditions were obtained using a sample pH of 4.0, a sample flow rate of 6.0 mL min^−1^, 30.0 mg of sorbent mass, and 1.0 mol L^−1^ HNO_3_ as the eluent at a flow rate of 4.0 mL min^−1^. The limit of detection for zinc was 1.9 *μ*g L^−1^, and the precision was below 0.82% (20.0 *μ*g L^−1^, *n* = 7). The analytical curve was linear from 2 to 50 *μ*g L^−1^, with a correlation coefficient of 0.9996. The method developed was successfully applied to spiked Brazilian sugar cane spirit, and accuracy was assessed through recovery tests, with results ranging from 83% to 100%.

## 1. Introduction

Brazilian sugar cane spirit (*cachaça* in Portuguese) has received growing attention since it is increasingly being appreciated worldwide [1]. This alcoholic beverage is obtained from the distillation of the sugar cane fermented must (wine) employing basically two types of apparatus homemade copper pot stills (alembics) or industrial stainless steel columns. 

Minas Gerais State is by far the largest producer of high-quality artisanal *cachaça* in Brazil, and the exportation of this product has reached significant levels of economic importance. The production of artisanal *cachaça* is also directly related to other important economic activities such as the production of cow's milk, beef, and organic fertilizer, since the sugar cane residues (leaves and tips), as well as the tail distillation fraction (known as *vinhoto*), can be used as cattle feed in the dry season, when appropriate pasture land becomes scarce [2]. 

However, less than 1% of the volume produced is exported. Efforts have been made to increase the export volume and qualify *cachaça* as an international Brazilian beverage [3]. Great improvements have been made regarding the determination of the chemical composition of *cachaça* in the past decade [4–8]. Consequently, quality control has been improved, and producers are also able to successfully control the chemical composition and sensory profile.

Metal elements in distilled beverages come from the raw materials, crop treatment, or manufacturing processes [9]. Thus, knowledge of the inorganic profile of Brazilian *cachaça* is important for the control of the heavy metal ion concentrations, thereby contributing to the improvement of the beverage quality.

Analytical methods for metal determination frequently require sample preconcentration and/or pretreatment for the destruction of the organic matrix, such as wet digestion, dry ashing, and microwave oven dissolution [10, 11]. Methods commonly employed include atomic absorption spectrometry (AAS), atomic emission spectrometry (AES), and inductively coupled plasma-optical emission spectrometry (ICP-OES) [12]. Ion chromatography is also used for the analysis of metals, for example, in vodka [13]. Unfortunately, most of these methods involve rather expensive instrumentation (associated with high-cost maintenance) which precludes their widespread use by alcoholic beverage producers [14].

However, preconcentration methods based on solid phase extraction are attractive when coupled online with the detection instrument and with the use of a high sorption capacity sorbent, such as *Moringa oleifera* seeds.

*M. oleifera* is the best-known species of the* Moringaceae* family. It is a plant native to northwest India which has spread all over the world, mainly in tropical countries. *M. oleifera* seeds have been used for the treatment of turbid water due to their flocculation properties [15]. The *M. oleifera *husks have been used for the production of activated carbon of high quality and microporosity [16]. The manufacturing procedures have been simplified to allow the production of carbon products of much lower cost than those available on the market. Since it is a natural ion exchanger, these materials can be used as biosorbents in solid-phase extraction processes.

According to Brazilian legislation, *cachaça* can have a maximum of 5 mg L^−1^ of zinc, and this demonstrates the importance of developing a simple, fast, and relatively low-cost methodology to control the quality of products manufactured in an artisanal way.

Thus, due to the possible sources of contamination by metal ions, such as zinc, during the production of alcoholic beverages, the objective of this study was to develop a methodology for an online preconcentration system, using activated carbon obtained from *M. oleifera* husks as a biosorbent, coupled to flame atomic absorption spectrometry (FAAS), for the determination of zinc in Brazilian sugar cane spirit samples.

## 2. Experimental

### 2.1. Instrumentation

A Varian Model SpectrAA 220 (Victoria, Australia) flame atomic absorption spectrometer equipped with a zinc hollow cathode lamp and a deuterium lamp for background correction were used for the detection of zinc. The instrument was operated under the conditions recommended by the manufacturer: lamp current of 5 mA, wavelength of 213.9 nm, slit width of 1.0 nm, burner height of 17 mm, acetylene flow rate of 2.0 L min^−1^, and air flow rate of 13.5 L min^−1^. 

The flow preconcentration system was constructed using a Gilson Minipuls 3 peristaltic pump (Villiers Le Bel, France) equipped with eight channels and Tygon and polyethylene tubes were used to pump the solutions through the minicolumn (60 mm ×  3 mm) in the elution and preconcentration steps. A Gehaka PG1800 pH meter was used to adjust the pH of the samples and working solutions.

A fourier transform infrared (FT-IR) spectrometer (Shimadzu, IRPrestige-21, Tokyo, Japan) was used to characterize the functional groups of activated carbon obtained from the husks of *M. oleifera* seeds.

### 2.2. Reagents, Solutions, and Samples

All working solutions were prepared with deionized water obtained from a Gehaka (São Paulo, Brazil) water purification system.

All reagents were analytical grade. Before use, laboratory glassware was kept overnight in 10% (v/v) nitric acid aqueous solution, followed by ultra-sonication for 1 h and finally rinsed with deionized water. Working solutions of zinc were prepared daily by appropriate dilution of a 1000 mg L^−1^ standard zinc solution (Carlo Erba, Val de Reuil, France). The nitric acid solution used as the eluent was prepared through dilution in water of concentrated nitric acid obtained from Merck (Darmstadt, Germany). 

Brazilian sugar cane spirit samples were purchased at local markets in Ituiutaba and Uberlândia (Minas Gerais State, Brazil) and analyzed without prior treatment.

### 2.3. Preparation of the Column

The *M. oleifera* seeds used to produce the activated carbon were obtained from trees cultivated in the city of Uberlândia (Minas Gerais, Brazil), washed thoroughly with deionized water to remove water soluble impurities, and dried at room temperature for 8 h.

The husks were separated from the seeds, crushed in a blender (Black & Decker, São Paulo, Brazil), and finally passed through 500 to 850 *μ*m sieves. The sieved material was rewashed thoroughly with deionized water to remove the fine particles, dried at 100°C for 4 h, and treated with 0.1 mol L^−1^ nitric acid and methanol for 4 h to remove inorganic and organic matter from the sorbent surface. Thermal treatment in an electric furnace (Cienlab, São Paulo, Brazil) at 200°C was then applied for 1 h to increase the surface area of the material [17]. The activated carbon obtained was then placed in a desiccator for later use as a biosorbent.

The minicolumns were comprised of polyethylene tubes with an inner diameter of 3 mm and were sealed at both ends with glass wool. The minicolumn (60 mm × 3 mm) was filled with 30 mg of the activated carbon, and the performance was stable during all experiments.

### 2.4. Online Preconcentration System

**Figure 1:**
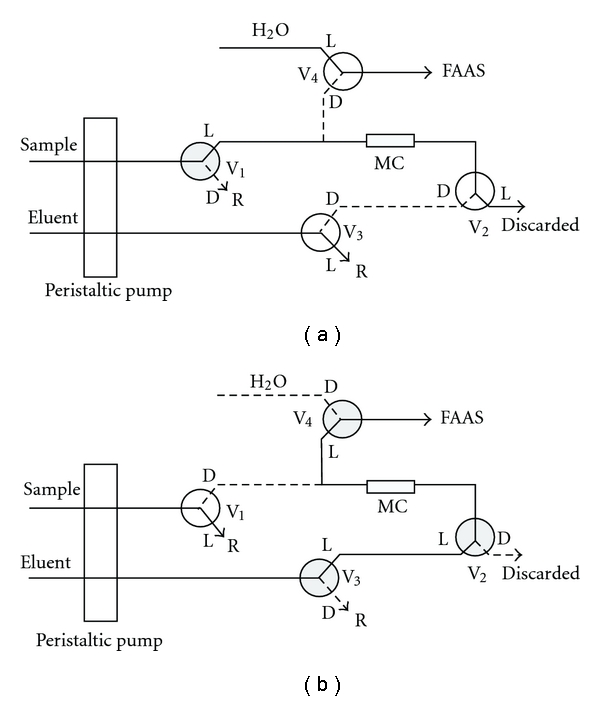
Diagram of the online preconcentration system used in this study. (a) Adsorption process and (b) desorption process. V, valve; L, open; D, closed; MC, minicolumn containing adsorbent; R, sample or eluent back stream; hatched circle, valve on; white circle, valve off.

The flow system consists of a peristaltic pump equipped with Tygon tubes, four three-way solenoid valves and a minicolumn filled with biosorbent. The diagram of the flow preconcentration system is displayed in Figure 1. At the preconcentration step position (Figure 1(a)), 10 mL of the sample at pH 4.0 were percolated through a minicolumn (30 mg) at 6.0 mL min^−1^ flow rate. During the preconcentration step (Figure 1(a)), valve 1 is open, and valves 2, 3, and 4 remain closed. After this stage, valve 1 is closed and valves 2, 3, and 4 are open, a stream of 1.0 mol L^−1^ HNO_3_ displaces the zinc ions at 4.0 mL min^−1^ flow rate (Figure 1(b)). Afterwards, the eluted zinc ions are pumped directly to the nebulization system of the FAAS.

### 2.5. Optimization System

The optimization of the parameters affecting the sorption of Zn by the activated carbon obtained from the husks of the *M. oleifera* seeds was performed using a two-level full factorial experimental design involving four factors and final optimization using a response surface. All experiments were carried out in duplicate, using 10.0 mL of 10 *μ*g L^−1^ Zn solution. The variables studied were sample pH, adsorbent mass, eluent concentration, and sample flow rate.

### 2.6. Interferences

In order to investigate the selective separation and determination of zinc ions from real samples containing different metal ions, a 10 *μ*g L^−1^ of zinc solution and increasing amounts of possible interfering ions were taken and submitted to the preconcentration procedure. In this study, interference was investigated using a 2^7−3^ fractional factorial experimental design including a central point, resulting in 17 experiments. The results were compared with zinc preconcentration in the absence of concomitant.

## 3. Results and Discussion

### 3.1. Characterization of Biosorbent

**Figure 2:**
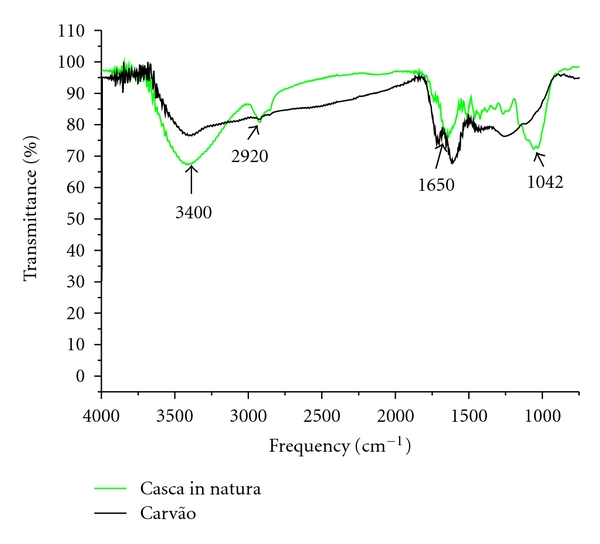
FT-IR spectra for *Moringa oleifera* husks and activated carbon.

The FT-IR technique was used to study the main functional groups of the activated carbon and *M. oleifera* husks, and Figure 2 shows the FT-IR spectra obtained.

The thermal degradation of husks occurs through their dehydration and the formation of CO and CO_2_ molecules, released in vapor form. The carbonization is characterized by the disappearance of chemical functional groups originally present in the precursor molecules and the formation of compounds with low molecular weight.

The most significant bands in the analysis of the activated carbon obtained were for lignocellulosic materials, present in the spectra at around 3400 cm^−1^, 2920 cm^−1^, 1730 cm^−1^, 1650 cm^−1^, and in the range of 1300–900 cm^−1^ [18].

For the husks, the precursor of the activated carbon, a broad band centered at 3400 cm^−1^ was assigned to O–H stretching, associated with the water absorbed on the surface of the material and silanol groups (SiOH). In the region of 2920 cm^−1^, there was a strong signal due to O–H stretching of the methyl groups, these groups being present in the structure of lignin [19].

Analysis of the differences between the spectrum of the pyrolyzed activated carbon at 200°C and that of the *in nature* husks shows the effect of heating on the chemical structure of the precursor material. The band at 3400 cm^−1^ is attributed to O–H vibration remains, but the C–H vibrations of methyl and methylene groups are no longer discernible, indicating a decrease in the aliphatic character of the material.

### 3.2. Optimization

Preliminary tests were performed to investigate which factors exert significant influence on the adsorption of Zn (II) by the activated carbon. The factors selected were eluent concentration, sample pH, adsorbent mass, and sample flow rate. Eluent type and eluent flow rate were fixed as a nitric acid aqueous solution at 1.0 mol L^−1^ and 4.0 mL min^−1^, respectively. These were selected based on preliminary studies [20], which took into account the absence of a carry-over effect, the background signal, and the shape of the transient signal obtained. 

Table 1 shows the response for each factorial design experiment. The analytical response was taken as the integrated absorbance, and the sample volume used for the preconcentration was 10 mL spiked at 10 *μ*g L^−1^ Zn.

**Figure 3:**
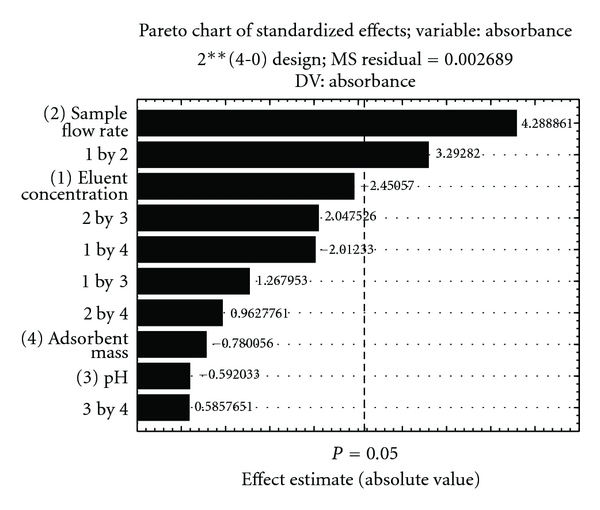
Pareto chart obtained from the optimization study of the variables, with their significance, for the preconcentration of Zn(II) using activated carbon obtained from *Moringa oleifera* husks as the sorbent and FAAS.

From the results reported in Table 1, a Pareto chart (Figure 3) was plotted to check the influence of the factors and their interactions in the system. An effect was considered significant when it was above the standard error at the 95% confidence level (*P* > 0.05), which is denoted by the vertical line on the graph.

The Pareto chart shows that within the range studied, the mass of the adsorbent and the pH showed no significant influence, and these parameters were thus kept at 30 mg and 4.0, respectively. The literature shows that to ensure interaction between the metal ion and the surface of the adsorbent, the pH must be such that M^n+^ is the most abundant metal ion species [20], and the sorbent surface is negatively charged, that is, above its point of zero charge. Above this pH, the sorbent surface is negatively charged, and the most abundant species of zinc is Zn^2+^. Thus, the interaction between the negative surface of the sorbent and the positive metal ion leads to the retention of the zinc in the proposed preconcentration system [30].

**Figure 4:**
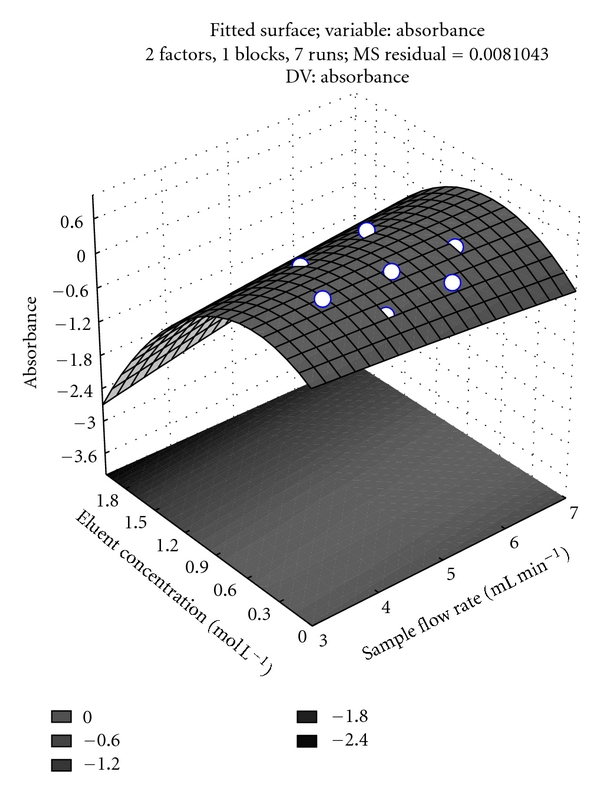
Response surface for optimization of eluent concentration and sample flow rate: sample volume, 10.0 mL; sample concentration, 10 *μ*g L^−1^; sorbent mass, 30 mg; pH, 4.0.

The most significant variables indicated by the factorial design (sample flow rate and eluent concentration) were then optimized using a response surface. The results were used to construct the surface response shown in Figure 4. The response surface can be described by the quadratic equation:

(1) $$\begin{array}{cc} \text{Abs} & =0.130-0.028\left(\text{sample flow rate}\right)\\ & \;+0.399\left(\text{eluent concentration}\right)\\ & \;+0.002{\left(\text{sample flow rate}\right)}^{2}\\ & \;-0.267{\left(\text{eluent concentration}\right)}^{2}\\ & \;-0.0168\left(\text{eluent concentration}\right). \end{array}\;$$

The critical points obtained were 1.0 mol L^−1^ and 6.0 mL min^−1^ for eluent concentration and sample flow rate, respectively. The application of the Lagrange criterion indicated that the critical point is the maximum point of the response surface.

Thus, as a result of all the optimizations, the following working conditions were selected: sample pH 4.0, 1.0 mol L^−1^ HNO_3_ as the eluent, and sample flow rate of 6.0 mL min^−1^.

### 3.3. Interference

**Figure 5:**
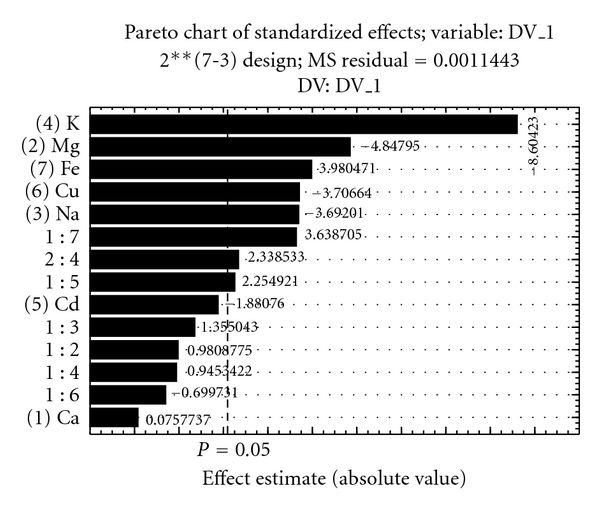
Pareto chart of effects of interfering ions on zinc adsorption in the proposed preconcentration system.

The effect of Ca^2+^, Mg^2+^, Na^+^, K^+^, Cd^2+^, Fe^3+^, and Cu^2+^ on the determination of zinc was studied using a mixed solution method, where the solution contained a fixed concentration of zinc and various concentrations of interfering ions. Solutions were prepared containing 10 *μ*g L^−1^ of zinc and 250 and 500 *μ*g L^−1^ of possible interfering ions. The solutions containing the zinc sample plus the potential interference ions were analyzed by the proposed method. The response was compared to that obtained from an uncontaminated zinc solution. From the results presented in Table 2, a Pareto chart was constructed (Figure 5). Metal ion was considered interfering when it was above the standard error at 95% confidence level (*P* > 0.05), which is denoted by the vertical line on the graph.

This study suggests that all concomitant ions were significant (*P* > 0.05), except Ca^2+^ and Cd^2+^. These interferences can be attributed to competition from concomitant ions by adsorptive sites, since the interaction of metal species on the adsorbent surface occurs primarily by ion exchange or complexation. However, the levels of concomitant ions in the real samples are much lower than the level studied in this research.

### 3.4. Analytical Features

With the optimized system, the method was evaluated through the main analytical features. A good correlation coefficient was obtained (0.9996) between the analytical signal predicted by the linear function and the analytical signal observed experimentally in the linear range of 2–50 *μ*g L^−1^. The repeatability of the proposed method was assessed by performing seven consecutive extractions at a concentration level of 20 *μ*g L^−1^ and expressing the result in terms of the relative standard deviation. A value of 0.82% was obtained demonstrating an excellent repeatability. Therefore, the preconcentration factor (PF), defined as the ratio of the slopes of the linear equation of the calibration graphs before and after the preconcentration, was 10.9. The limit of detection (LOD) was calculated as 3*σ*/S, where S is the slope of the calibration curve and *σ* is the standard deviation of 10 consecutive measurements of the blank solution and the value of the LOD was 1.9 *μ*g L^−1^.

### 3.5. Application of the Method and Recovery Tests

The proposed method was applied to five Brazilian *cachaça* samples. In all samples the analyte concentration was below the limit of detection of the method. Thus, in order to assess the analyte recovery, all the samples were spiked at concentration levels from 0 to 40 *μ*g L^−1^, and analytical curves were constructed in order to compare the slopes. The results obtained are shown in Table 3, where it can be seen that there is no difference between the recovery values for the samples, indicating that the analyte is quantitatively retained in all of the samples evaluated. The recovery values provided evidence of the good accuracy of the method. 

In the present study, the method proposed for determining zinc in beverage samples was described and compared according to the detection technique used. The most important details of the published procedures for zinc determination, in terms of kind of sample, are presented in Table 4. Most of the methods were applied to the analysis of zinc in water (natural, seawater). To compare the method studied in this paper, the limit of detection, preconcentration factor, and sample volume have been taken into account. The direct methods provide the lowest limit of detection (about 6.0 *μ*g L^−1^) [21]. However, direct methods involve matrix matching calibration and present more important interferences derived from the presence of ethanol. For this reason, these methods often require tedious pretreatments of the sample that will vary if is a solid or liquid sample and depend on the concentration of the possible interference substances. The electroanalytical methods provided limit of detection about 10 *μ*g L^−1^ [22]. These methods present potentially interference substances that are substances formed by different anions of organic acids, customarily existent in the analyzed samples, producing negative peak signals. Preconcentration methods based on solid-phase extraction are attractive when coupled with the detection instrument and with the use of sorbent and chelating agents. These methods provided limit of detection about 0.2 to 29 *μ*g L^−1^ and preconcentration factor about 5 to 144 [23–29]. As observed, the adsorbent preconcentration method presented here shows better or similar performance when compared with the works previously published in terms of limits of detection and sample consumption. The procedure developed based on a natural adsorbent with FAAS detection allowed for the determination of zinc at the level of *μ*g L^−1^ in Brazilian *cachaça *without the need for a specific sample preparation step.

## 4. Conclusions

The proposed method represents an alternative low-cost procedure for the determination of zinc in Brazilian *cachaça* samples, compared to commonly used methods. It was shown to be appropriate for the rapid and accurate quantification of zinc and does not require pretreatment of the samples. Furthermore, it involves the use of flame atomic absorption spectrometry, a simple technique of easy operation, which has low operational and maintenance costs compared with other absorption or emission techniques.

## Figures and Tables

**Table 1:** Conditions for preconcentration of zinc and analytical response for the study of multivariate optimization using SPE with detection by FAAS.

Run	Sorbent mass (mg)	pH	Sample flow rate (mL min^−1^)	Eluent concentration (mol L^−1^)	Integrated absorbance
1	30	4.0	3.0	0.5	0.4473
2	60	4.0	3.0	0.5	0.4806
3	30	9.0	3.0	0.5	0.3160
4	60	9.0	3.0	0.5	0.3926
5	30	4.0	6.0	0.5	0.4136
6	60	4.0	6.0	0.5	0.4430
7	30	9.0	6.0	0.5	0.4473
8	60	9.0	6.0	0.5	0.4358
9	30	4.0	3.0	1.5	0.3780
10	60	4.0	3.0	1.5	0.1696
11	30	9.0	3.0	1.5	0.2877
12	60	9.0	3.0	1.5	0.2055
13	30	4.0	6.0	1.5	0.4236
14	60	4.0	6.0	1.5	0.4277
15	30	9.0	6.0	1.5	0.4894
16	60	9.0	6.0	1.5	0.4864

**Table 2:** Resulting matrix of the factorial design and analytical response*.

Run	Ca^2+^	Mg^2+^	Na^+^	K^+^	Cd^2+^	Fe^2+^	Cu^2+^	Integrated absorbance
1	0	0	0	0	0	0	0	0.2802
2	500	0	0	0	500	0	500	0.2471
3	0	500	0	0	500	500	0	0.0689
4	500	500	0	0	500	500	500	0.1440
5	0	0	500	0	0	500	500	0.1385
6	500	0	500	0	0	500	0	0.0709
7	0	500	500	0	500	0	500	0.0980
8	500	500	500	0	0	0	0	0.0821
9	0	0	0	500	500	500	500	0.0779
10	500	0	0	500	500	500	0	0.0120
11	0	500	0	500	0	0	500	0.0509
12	500	500	0	500	0	0	0	0.0137
13	0	0	500	500	0	0	0	0.0000
14	500	0	500	500	0	0	500	0.1234
15	0	500	500	500	500	500	0	0.0000
16	500	500	500	500	500	500	500	0.0286
17	250	250	250	250	250	250	250	0.0000

*Concentration of each interfering ion is given in *μ*g L^−1^.

**Table 3:** Experimental recovery for determination of zinc in Brazilian *cachaça* samples by online preconcentration method.

Sample	Zn (*μ*g L^−1^)		Recovery (%)
	Added	Found*	
1	10.0	9.2 ± 0.1	92.5
	40.0	39.9 ± 0.5	99.9
2	10.0	10.8 ± 0.2	108.4
	40.0	39.2 ± 0.4	97.9
3	10.0	10.3 ± 0.2	103.1
	40.0	39.9 ± 0.5	99.8
4	10.0	9.0 ± 0.1	90.4
	40.0	37.4 ± 0.5	93.6

*Results are expressed as mean values ± S.D. based on three replicate (*n* = 3) determinations. Confidence interval, 95%.

**Table 4:** Comparison of methods for determination of zinc.

Sample	Sorbent	Chelating agent/modifier	Eluent	PF	SV (mL)	LOD (*μ*g L^−1^)	Linear range (*μ*g L^−1^)	Detection	Ref.
Brazilian *cachaça *	—	—	—	—	—	6.0	0–4000	FAAS	[21]
Sugar	—	—	—	—	—	10	25–200	ASV	[22]
Water, hair, urine and saliva	Alizarin Red S	Alumina	HNO_3_	144	25	0.2	1–100	FAAS	[23]
Seawater	Silica gel	DPTH	Citric acid/tartaric acid	—	8.8	1.7	2–500	ICP-OES	[24]
Water	Amberlite XAD-2	oVTSC	HCl	140	1000	10	20–50	FAAS	[25]
No data	XAD-7	8-BSQ	HCl	10	—	1.6	5–200	Spectrophoto-metry	[26]
Sea water	Dowex 1X8-50	ARS	HNO_3_	5	50–200	23	No data	FAAS	[27]
Saline matrices	Amberlite XAD-7	ARS	HNO_3_	50	50	29	250–2000	FAAS	[28]
Water	SiO_2_/Al_2_O_3_/ Nb_2_O_5_	PAN	HNO_3_	52.6	20	2.3	30–180	Spectrophoto-metry	[29]
Brazilian *cachaça *	*M. oleifera* husks	—	HNO_3_	10.9	10	1.9	2–50	FAAS	This work

PF = preconcentration factor; SV = sample volume; LOD = limit of detection; PAN = (1-(2-piridylazo)-2-naphtol); DPTH = 1,5-bis(di-2-pyridyl)methylene thiocarbohydrazide; ARS = Alizarin red S; OVTSC = vanillin thiosemicarbazone; 8-BSQ = 8-(benzenesulfonamido)quinoline; FAAS = flame atomic absorption spectrometry; ASV: anodic stripping voltammetry, ICP-OES = inductively coupled plasma optical emission spectrometry.
